# Endovascular Treatment With a Balloon-Expandable Covered Stent in a Polytrauma 12-Year-Old Patient With Traumatic Abdominal Aortic Rupture

**DOI:** 10.7759/cureus.63135

**Published:** 2024-06-25

**Authors:** Maria Florou, Chrysostomos Kepertis, Kyriakos Stavridis, Manolis Abatzis-Papadopoulos, Maria Tsopozidi, Kleanthis Anastasiadis, Konstantinos Tigkiropoulos, Vassileios Mouravas

**Affiliations:** 1 2nd Department of Pediatric Surgery, Aristotle University of Thessaloniki, Papageorgiou General Hospital, Thessaloniki, GRC; 2 Department of Vascular Surgery, Papageorgiou General Hospital, Thessaloniki, GRC; 3 Department of Pediatric Surgery, Papageorgiou General Hospital, Thessaloniki, GRC; 4 1st Department of Surgery, Division of Vascular Surgery, Aristotle University of Thessaloniki, Papageorgiou General Hospital, Thessaloniki, GRC

**Keywords:** emergency, endovascular stent, pediatric, trauma, abdominal aorta

## Abstract

Βlunt trauma is a common injury in children; however, blunt abdominal aortic trauma is extremely rare and is accompanied by high rates of morbidity and mortality. We report our initial experience with the endovascular management of an abdominal aortic rupture in a 12-year-old boy after he was involved in a motor vehicle accident. The patient was a backseat-restrained passenger. Upon admission, he had a Glasgow Coma Scale of 15, was hemodynamically stable, and complained of abdominal pain. The computed tomography revealed a rupture in the abdominal aorta along with a distally extending pseudoaneurysm, free fluid in the peritoneal cavity, and a large retroperitoneal hematoma. The rest associated injuries were a Grade III splenic injury, a retroclival epidural hematoma in the first cervical vertebra level, a right clavicle fracture, a bilateral minor pneumothorax, along with bilateral pulmonary lacerations and contusions in the thoracic vertebrae. Given the extent of the intraabdominal injuries and the risk for open laparotomy, the decision to proceed with endovascular stenting instead of open surgical repair was made. The patient tolerated the procedure well and an angiography confirmed the result. The postoperative period was uneventful and the associated injuries were treated conservatively without complications. Although blunt abdominal aortic trauma is extremely rare in children, endovascular management seems to be a new and feasible therapeutic approach.

## Introduction

Blunt trauma is the most common injury in children and remains the most common cause of death and disability in the pediatric population. On the other hand, blunt trauma in the abdominal aorta is extremely rare and only a few cases have been reported in the literature [1,2]. Motor vehicle accidents and the associated seat-belt positioning remain the main causes of such injuries because children are quite vulnerable to trauma due to their particular anatomy and the failure to use an appropriately sized seat belt [3,4]. The abdominal aortic trauma is accompanied by high rates of morbidity and mortality, and prompt diagnosis and treatment should be applied [5]. Although endovascular, repair has become the standard approach in adults, in the pediatric population, the standard management includes either the open surgical repair or the conservative management, owing mainly to the smaller aortic vessel diameter and the lack of follow-up data [1,2]. To the best of our knowledge, this is one of the few papers presenting the endovascular management of a seat-belt-related abdominal aortic rupture with a balloon-expandable covered stent in a child.

## Case presentation

**Figure 1:**
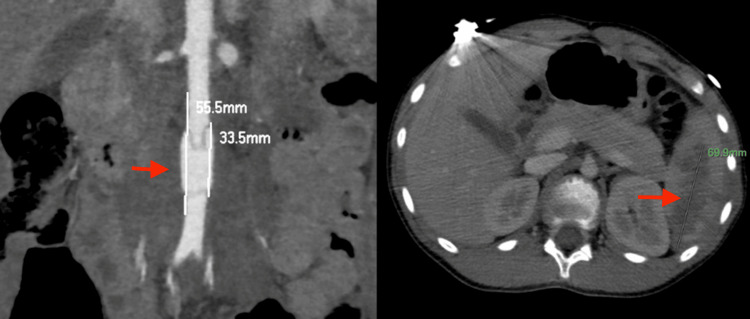
The abdominal aorta trauma just lower on the level of the inferior mesenteric vessels, along with a 3.3 cm-long pseudoaneurysm (pointer). Grade III splenic injury with an extended intraparenchymal hematoma in the lower splenic pole (pointer).

A 12-year-old boy presented to our emergency department with multiple injuries caused by a car accident at a high velocity. The patient was a backseat passenger restrained on the right side of the car. Upon admission, the boy had a Glasgow Coma Scale of 15, was hemodynamically stable, and complained of abdominal pain. Clinical examination revealed a painful and guarding abdomen with large, seat-belt-related bruises on the body. The computed tomography examination showed a rupture in the abdominal aorta just lower on the level of the inferior mesenteric vessels, along with a 3.3 cm-long distally extending pseudoaneurysm, free fluid in the peritoneal cavity, and a large retroperitoneal hematoma extending to the pelvis (Figure 1).

**Figure 2:**
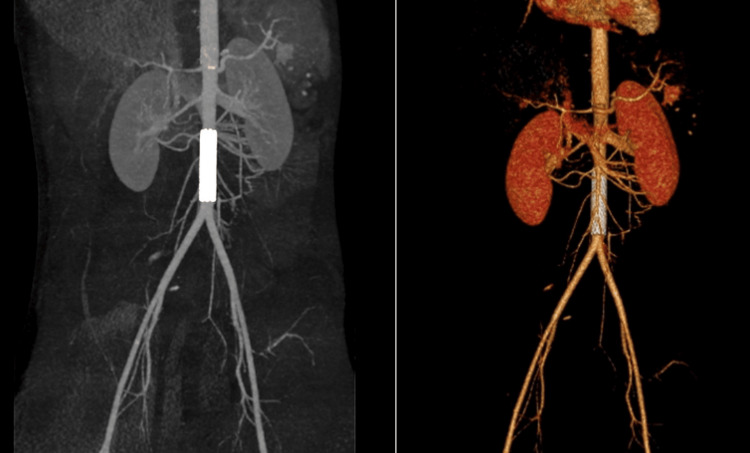
Postoperative computed tomography-angiography verifying the right placement of the endovascular aortic stent.

Furthermore, a Grade III splenic injury was revealed, with an extended intraparenchymal hematoma in the lower splenic pole approximately 7 cm in diameter (Figure 1). The rest of the associated injuries were a retroclival epidural hematoma on the level of the first cervical vertebra (C1), a right clavicle fracture, bilateral pulmonary lacerations, bilateral minor pneumothorax, and contusions in the thoracic vertebrae. Given the extent of the intraabdominal injuries and the risk for an open laparotomy, an urgent vascular surgical opinion was requested, and the decision to proceed with an endovascular stenting instead of an open, surgical repair was made. Under general anesthesia, the open exposure of the left common femoral artery was applied and a 7F-sheath placement followed. After confirming with intraoperative angiography that the wire was in the right position, the angioplasty balloon and the expandable covered Bentley stent (12x59 mm, BeGraft Aortic, Bentley InnoMed, Germany) were advanced. A confirmatory angiography followed. The patient tolerated the procedure well and he returned to the pediatric surgery department after a few hours in close monitoring. The postoperative period was uneventful and 2.000 International Units (IU) of low-molecular-weight heparin - 100 IU/kg of body weight - was applied subcutaneously as a prophylaxis due to stent placement, daily (Figure 2).

The patient remained hemodynamically stable and the routine repeat imaging showed slow and progressive improvement of the splenic injury, so the conservative management was continued. Likewise, the follow-up examinations of the retroclival epidural hematoma with magnetic resonance imaging showed stability and improvement, and along with the good neurological condition of the boy, it was decided by the neurosurgeons to observe and support the patient. The rest of the associated injuries, including the right clavicle fracture, the small pneumothorax, and the vertebrae contusions, were also treated conservatively without any complications. The recovery was slow, and the boy started oral feeding on the fourth postoperative day uneventfully. He had a prolonged hospitalization and he was discharged on the 19th postoperative day with a prescription of acetylsalicylic acid in an 80 mg daily dose, oral intake.

## Discussion

Blunt abdominal aortic trauma is a very rare occurrence in children, accompanied by significant morbidity and mortality. It has an approximate incidence of 0.05% in pediatric trauma patients who have survived the pre-hospital phase after trauma, and thus it is considered underestimated, as a great majority of these injuries are fatal at the moment of the accident [2,3]. Motor vehicle accidents are the leading cause, accounting for 65% of these lesions in the pediatric population. Falls and child abuse incidents are the other usual mechanisms of this injury [2]. Children are often restrained passengers, wearing lap belts or lap-shoulder belts, who are involved in high-speed deceleration road accidents [4]. They are quite vulnerable to seat-belt-related trauma due to their particular anatomy, increased head-to-body ratio, small body size, and wrong lap-belt positioning [5,6]. The associated injuries are very common because pediatric aortic trauma is the result of the application of tremendous force. As a result, pediatric abdominal aorta injuries have been reported in the context of seat-belt syndrome [6,7]. The syndrome encompasses a combination of lesions: linear ecchymoses on the abdominal wall skin, intraabdominal solid organ injuries, vertebral column fractures, and intestinal injuries. The involvement of the aorta may change the term to the so-called seat-belt aorta [6,8]. Similar to this description, our patient presented with a rupture in the abdominal aorta, along with a splenic injury, vertebral column contusions, a clavicle fracture, and head and thorax injuries. 

According to the literature, abdominal aortic trauma is accompanied by a 7.5% in-hospital mortality rate and 22.5% of residual sequelae in the pediatric population, a fact that necessitates prompt diagnosis and management. Computed tomography, with or without an angiography has traditionally been the gold-standard examination for evaluating a polytrauma patient [5]. Regarding the management, no consensus guidelines have been reported on the optimal treatment of children with blunt abdominal aorta trauma [2]. Two therapeutic approaches have been described in the literature, including either conservative management with intravenous resuscitation and repeat imaging or open surgical repair of the aortic trauma for hemodynamically unstable patients. Endovascular therapy has become widely accepted during the last two decades in adults. In the pediatric population, the endovascular stent placement has been more widely described for blunt injuries of the thoracic aorta, rather than the abdominal aorta. There are very few literature reports describing the procedure for the abdominal aorta. The first presented case of successful endograft repair of abdominal aorta transection was in a 10-year-old patient in 2006 [9]. The patient was followed up for four years, uneventfully, with no adverse effects with regard to the child’s growth [2]. Since then, there have been scattered case reports and case series describing the endovascular management of abdominal aortic trauma with satisfactory results, increasing the awareness of this technique and offering a probable new, feasible, and safe therapeutic approach [4-6,10]. 

On the other hand, the difficulties associated with small vessel size in the pediatric population and the lack of long-term outcomes highlight the necessity to be watchful. Since pediatric patients are a growing population, there is a great need for data reassuring that endovascular grafting has good results as children thrive and has good long-term results in adult life [1,2]. Our patient was discharged with instructions for repeat imaging with computed tomography and angiography as soon as he enters adolescence and adulthood. 

## Conclusions

Blunt abdominal aortic trauma is a very rare occurrence in the pediatric population and it is accompanied by high rates of mortality and morbidity. The standard management has included either the conservative treatment or the open surgical repair so far. The endovascular management of abdominal aortic trauma seems to be a new and feasible therapeutic approach for polytrauma pediatric patients. This is one of the very few reports discussing the endovascular treatment of an abdominal aortic rupture with an expandable covered stent in children. However, the literature data are limited to case reports and case series, recording short-term results so far. More studies are needed to follow up on the children as they thrive and evaluate the long-term outcomes of endovascular aorta management.
